# The determinants of anti-diabetic medication adherence based on the experiences of patients with type 2 diabetes

**DOI:** 10.1186/s13690-019-0347-z

**Published:** 2019-05-16

**Authors:** Laleh Dehdari, Tahereh Dehdari

**Affiliations:** 1 Department of Statistics, School of Mathematics Sciences, Yazd University, Yazd, Iran; 2 Department of Health Education and Health Promotion, School of Public Health, Iran University of Medical Sciences, Shahid Hemmat Highway, Tehran, Iran

**Keywords:** Medication adherence education, Experiences, Qualitative research, Diabetic patients

## Abstract

**Background:**

The purpose of this study is to explain the experiences of patients with type 2 diabetes (T2DM) about medication adherence.

**Method:**

A qualitative content analysis study was conducted at the Institute of Endocrinology and Metabolism Research and Training Center of Firoozgar Academic Hospital, affiliated to Iran University of Medical Sciences in Tehran, Iran during April–June 2017. Twenty-six semi-structured in-depth interviews were conducted with diabetic patients and their families. The participants were asked about their experiences of medication adherence. After the first round of the interviews, content analysis of data started and continued up to the data saturation.

**Results:**

Four main themes including perceived barriers (with 6 subthemes including inadequate knowledge, situational influences, inadequate perceived threat about diabetes, treatment characteristics, personality traits and medication cost), perceived social support (with 3 subthemes including family, doctor and community supports), medication beliefs (with 3 subthemes including belief in the effectiveness of treatment, belief in the more effectiveness of complementary therapies than medication use and prioritizing the use of the pills instead of the insulin injection) and cues to action (with 2 subthemes including internal and external cues to action) emerged as the experiences of the participants in terms of anti-diabetic medications adherence which should be considered in developing medication adherence interventions for the patients with T2DM.

**Conclusion:**

The patients with T2DM had more barriers for regular drugs consumption. They had incorrect beliefs about anti-diabetic drugs. In addition, they were in need of abroad support community, surroundings and also health professionals. Health systems should consider medication adherence training to be added to the treatment protocols of the diabetic patients.

## Background

Type 2 Diabetes is a growing public health issue in Iran. Approximately 24% of Iranian adults above 40 years old suffer from Type 2 diabetes. The risk of the disease in Iranian women is 1.7% greater than men [1].

Patients with T2DM need to receive continuous medical care besides self-management and lifestyle modification education to prevent and reduce the risk of acute and long-term complications. Drug therapy is an essential element of diabetes care [2]. Successful glycemic control in type 2 diabetes requires the effective use of the prescribed medicines over time [3]. Several different pharmacological agents such as Metformin, Glinides, α-Glucosidase inhibitors, Insulin, Thiazolidinediones and Sulfonylureas are prescribed for glycemic control in patients with T2DM [4]. Although these agents can substantially reduce the diabetes-related morbidity and mortality, the extent of treatment benefits may be limited by a poorer prescribed medication adherence [3]. Non- adherence to chronic diseases has been described as taking < 80% of the prescribed treatment. Adherence to diabetes treatment has been reported to be suboptimal ranging from 23 to 77% [5]. Donnan et al. showed that one in three people with Type 2 diabetes had suitable adherence to prescribed oral hypoglycaemic medications [6]. The rate of non-adherence among Iranian patients with T2DM for using metformin, glyburide and insulin was 39.7, 35.3 and 28.8% respectively [7, 8]. In diabetic patients, poor adherence to medications was associated with a higher HbA_1C_ profile; more manifestation of diabetes-related complications such as retinopathy, neuropathy, and nephropathy; poor quality of life; greater healthcare costs; more prior primary care contacts and higher morbidity [7, 9, 10].

Poor medication adherence among people with diabetes is a complex behavior which can be caused by many factors such as being too busy; travelling; skipped meals; stress/emotional problems; public embarrassment; depression; medication cost; memory; knowledge, attitudes and beliefs about diabetes; worrying about side-effects of diabetes medicines; social support; interaction with health care providers; complexity of medication regimen; duration of the illness; concern about the negative effects of medicines and the presence of glucometer at home [8, 11–14]. However, additional information regarding determinant factors that influence medication adherence is required to develop tailored and effective interventions to promote medication adherence in patients with T2DM [15]. Therefore, given the lack of a comprehensive research on adherence-related factors among patients with T2DM, the objective of the present study was to explore the experiences of patients with T2DM in terms of medication adherence using a qualitative content analysis design.

## Methods

### Design

In this study, a qualitative content analysis design (conventional method) was used to analyze the experiences of Iranian patients with T2DM regarding the prescribed medication adherence.

### Setting and study participants

In the present study, purposive sampling (those who consumed anti- diabetes medications for at least one year and have not diabetes-related complications such as retinopathy, neuropathy, and nephropathy were included in the study) was used to recruit participants from the Institute of Endocrinology and Metabolism Research and Training Center of Firoozgar Academic Hospital, affiliated to Iran University of Medical Sciences in Tehran, Iran during April–June 2017. In this study, inclusion criteria were history of diabetes and blood glucose–controlling medications consumption at least for one year, not having diabetes-related complications such as retinopathy, neuropathy, and nephropathy, agreement to participate in study and their willingness to share and transfer direct and indirect experiences about the medication adherence. Exclusion criteria were defined as at each stage of the interview, those participants who did not wish to continue the interview could be excluded. In the study, no participant was excluded from the study.

### Data collection

Data saturation (i.e. when no new information is discovered in the data analysis) occurred by conducting 22 interviews with patients and 4 with their families. All interviews were recorded via audiotapes and transcribed verbatim. On average, each interview lasted approximately between 20 and 25 min. The interviews were conducted in a privacy room at the Institute of Endocrinology and Metabolism Research and Training Center of Firoozgar Academic Hospital. The decision about determining the time of conducting the interviews was made with the participants’ consent. Family members and the patients were interviewed separately. Interview topics included the participants’ and their family’s experiences of medication adherence, and influencing factors involved in irregular prescribed medication use. Sampling continued until saturation was reached. Demographic characteristics of the study participants included age, sex, marital status, occupation status and education level which were collected by means of a self-administered questionnaire.

### Data analysis

The interviews were recorded and next transcribed verbatim. The procedures involved in content analysis consisted of careful reviewing of the transcripts for four times. Upon reviewing the transcripts, a general impression was obtained. Then, based on the participants’ and their family members’ statements meaning units and their coding were conducted. Two researchers identified the initial codes in the data. Later, their coding was investigated for the degree of go-togetherness. Through further reviewing of the transcripts, initial codes were merged to account for the major themes in the data. In fact, through this data reduction procedure text was reduced to codes and themes. The emerging categories were constantly compared across and within the interviews to establish and refine categories which reflected the nuances of the data. Data collection proceeded in this way until saturation was achieved [16].

### Rigor

Since the participants refereed to the clinic for visit endocrinologist regularly several times in a year, the researchers had sufficient time to communicate and build trust and rapport with them. Prolonged engagement with the participants helped to achieve in-depth data collection. Maximum-variation sampling was employed based on the participants’ age, gender, education level and number of years with diabetes. To ensure that the analysis reflected the participants’ experiences accurately, member check was carried out with 11 participants and 2 of their family members. By using the external check method, two research advisors reviewed a random selection of more than 50% of the transcripts and the codes [17].

### Results

Twenty-two participants and four members of their families were interviewed. The demographic characteristics of the study participants are presented in Table 1. After data analysis, four main themes were identified: (a) perceived barriers, (b) medication beliefs, c) cues to action and d) perceived social support. Each of the subthemes was next divided into several other subthemes (Table 2).

**Table 1:** Descriptive statistics of the participants’ characteristics (*n* = 22)

Variables	*n*	*%*	*Mean*	*SD*
Age			56.72	9.16
Sex				
Female	12	54.5		
Men	10	45.5		
Marital status				
Single	1	4.5		
Married	20	90.9		
Divorced	1	4.5		
Education level				
Illiterate	0	0		
**≤** 12 ^th^ grade	19	86.4		
< 12th grade	3	13.6		
Occupational status				
Self-employed	5	22.7		
Employee	6	27.2		
Household duties	11	50		
The number of years of diabetes			9.30	7.40

**Table 2:** Categorization of Themes and Subthemes

I. Perceived barriers to medication adherence	
A. Medication cost	1. High cost of insulin 2. High doctor visits fees 3. High cost of glucose monitor test strips 4. High cost of psychological consulting classes
B. Treatment characteristics	1. High number of prescribed medications 2. Taking medications several times a day 3. Worrying about the side-effects of diabetes medicines 4. Long period of treatment 5. Increasing the medication dose over time
C. Personality traits	1. Not responsible 2. No respect for oneself and others 3. Attracting the pity and attention of family 4. Anxiety and restlessness
D. Situational influences	1. Party 2. Traveling 3. Early morning 4. Outdoors 5 Watching TV 6. Shopping 7. A gap between buying medications again 8. Irregular meals
E. Inadequate knowledge	1. Lack of knowledge about diabetes 2. Lack of knowledge about the effectiveness of medication treatments for the control of diabetes
F. Inadequate perceived threat about diabetes	1. Not accepting the diabetes as a disease 2. Not taking the diabetes seriously
II. Perceived social support	
A. Family support	1. Reminding the time of taking medications 2. Preparing the medicines 3. Recalling the complications of diabetes 4. Helping to follow-up the disease 5. Understanding the patient 6. Helping with insulin injection
B. Doctor support	1. Eliminating the patient’s fear of insulin 2. Being highly responsible for the control of diabetes 3. Being able of correct diagnosis 4. Giving morale to the patient 5. Spending time and responding patient’s questions 6. Appropriate behavior with the patient 7. Encouraging the patient to take regular medication 8. Informing about the complications of the irregular use of the medications 9. Describing the relationship between the medication and disease 10. Writing multiple prescriptions with more medication simultaneously
C. Community support	1. Giving information through mass media 2. Supporting to provide the medications 3. Increasing the insurance coverage
III. Medication beliefs	
A. Belief in the effectiveness of treatment	1. Belief in controlling diabetes by regular medication use 2. Belief in the effectiveness of taking the pills for controlling the blood glucose 3. Belief in the effectiveness of insulin injection than the pills use in controlling blood glucose
B. Belief in the more effectiveness of complementary therapies than medication use	1. Belief in the effectiveness of exercise than medication use in controlling the level of blood glucose 2. Belief in the effectiveness of having proper nutrition than insulin use in controlling the level of blood glucose 3. Belief in the effectiveness of herbal medications than modern medications in controlling the level of blood glucose
C. Prioritizing the use of the pills instead of insulin injection	1. Having pills being easier than the insulin injection 2. Pills being cheaper than insulin 3. Fear of needle and insulin injection 4. Difficulty with insulin injection 5. Decreasing morale due to insulin injection 6. More negative social stigma to insulin use 7. Existing difficult cconditions for keeping insulin outdoors 8. Negative family insight about the insulin injection 9. Feelings of disease progression following the insulin injection 10. Having good feeling about the use of the pills instead of insulin injection 11. Predicting death following the insulin injection 12. Appearing diabetes complications (such as kidney loss) following the insulin injection
IV. Cues to action	
A. Internal cues	1. Habit of taking medication 2. Fear of diabetes-related complications
B. External cues	1. Setting the alarm to remind the time of medications use 2. Presence of medication in front of the eyes

#### Themes

##### Perceived barriers

Perceived barriers, with six subthemes including inadequate knowledge, situational influences, inadequate perceived threat, treatment characteristics, personality traits and medication cost were identified as one of the variables that influenced the medication adherence among the participants.

From the participants^’^ point of view, high cost of anti-diabetic medication may decrease the patients^’^ medication adherence. Expensive insulin, doctor’s visit fees and blood glucose test strips as well as the lack of insurance were factors that greatly affected the patients’ compliance in their drug regimens.

*“There are lots of costs for a diabetic patient. He/she needs drugs. He/she needs insulin. Doctor visits or counseling sessions is very expensive! Access to drugs is very hard. Each time I pay about 13 or 14 US Dollars for the drugs. Some cannot buy! They do not have insurance or income. Although I can buy the drugs, sometimes it is hard for me to buy them. There should be a center to support us for buying the drugs” (A* 61-*year*-*old female*)

*“Glucose monitor tests strips is expensive. My nephews buy it for me. Glucometer is also very expensive. Because of that many do not continually check their blood glucose” (A* 59-*year*-*old female*)

Apparently such individual barriers inhibiting the regular medicines use included treatment regimen characteristics. In this category, factors such as high number of prescribed medications; taking medications several times a day; worrying about side-effects of diabetes medicines; long period of treatment and increasing the medications dose over time may affect the patients^,^ medication adherence.

*“Many are afraid of the side-effects of pills or insulin. They say it may affect kidneys. Also, pills may decrease the weight. I, for example, lost 19 kilos!” (A* 50-*year*-*old female*)

*“It’s overwhelming for me! Because I have many pills, and I have to eat them for several consecutive years! Doctor increases the pills each year. Its doses increase each year. It has increased from one to three. The number is boring!” (A* 63-*year*-*old male*)

Some participants and their families stated that four personality traits including being not responsible, no respect for yourself and others, attracting pity and attention of the family and anxiety and restlessness may influence on reducing the participants^’^ medication adherence.

*“If somebody cares and be responsible, he/she eats his/her drugs. Similar to other foods and plans, the time of having drugs should be set. Some patients do not care about what might happen to them” (A* 55-*year*-*old female*)

*“Patients should always keep the drugs with themselves. Anybody who respects him/herself, loving bodies and his/her health, consumes the drug orderly. Some patients stimulate others’ sense of pity to receive more attention! So, they don’t have their drugs to attract the attention of others” (A 30-year-old member of one female patients’ family).*

*“Stressful patients and those who suffer from depression and anxiety are more likely not to eat their drugs!” (A* 63-*year*-*old male*)

Many participants, both male and female, seemed to be strongly influenced by situational influences for regular use of their medications. Being at a party, traveling, the necessity to take the drugs early morning, being outdoors, watching TV, go shopping, the gap between buying drugs again and removing one of the meals were situational contexts which might interfere with the medications adherence among the participants.

*“When on a trip or at a party, my father does not eat his pills. He intentionally does it. I do not know why! Diabetic patients are like that. He does not like to eat pills outside. He feels exhausted by eating it. It makes no difference for them” (A 25-year-old member of one male patients’ family)*

*“I get up late! I can’t get up early! I feel dizzy. If getting up late, I forget to eat my pills or inject insulin. In fact, irregularity of food meals causes irregularity in having the pills. Eating pills with empty stomach bothers me!” (A* 56-*year*-*old female*)

*“One time I forgot to take my insulin out with myself and forgot to inject it. Being outdoors for shopping or any other reason causes the consumption disorder” (A* 62-*year*-*old female*)

*“The interval between buying the drugs and getting the renewed prescription from physician causes delay and stops in using my pills. It has been one week that I do not have the blood glucose pill. Now, I’ve come to get the prescription. I did not have time to come within the last week! ”(A 59*-*year*-*old female*)

Also, the participants in this study showed insufficient knowledge about diabetes and benefits of glucose-lowering medication for its control.

*“Lack of information about the illness causes irregular use of the drugs. Most of the patients who do not have enough information about the benefits of insulin or the pill do not like to eat or inject it” (A* 50-*year*-*old female*)

Subjects identified inadequate perceived threat as a barrier for the regular drugs use. They stated that some of diabetic patients do not accept diabetes as a disease and do not take diabetes seriously.

*“Due to lack of surface manifestations at early stages of diabetes, patients do not take it seriously. Especially, they do not eat drugs at early stages of diagnosis” (A 38*-*year*-*old member of one female’s patient’s family)*

*“I did not take seriously the first years of diabetes. I did not consider it as a disease. As my glucose lowered, I thought I had cured completely. Then, when I found its side-effects, I tried to use the pills orderly” (A* 56-*year*-*old female*)

##### Perceived social support

Acquiring support of family, doctor and community was very important for many patients because they helped in the treatment adherence and their illness control. The participants frequently indicated that families should support patients by reminding the time of taking medications to patients, preparing the medicines, recalling the complications of diabetes, helping to follow-up the disease, understanding the patient and helping with insulin injection. Also, they stated that doctors should reduce the patient’s fear of insulin, be highly responsible regarding diabetes control, have the ability of correct diagnosis, give morale to the patient, spend time and respond to patient questions, have appropriate behavior with the patient, encourage the patient to take regular medication, inform about the complications of the irregular use of medications, describe the relationship between medication and disease and write multiple prescriptions with more medication simultaneously. Also, giving information through mass media, support to provide the medications and increasing the insurance coverage were among the patients^’^ expected supports of their community.

*“Family should regularly remind the illness side-effects, orderly use of pills and drugs. They should care about the patient. They can also help in providing the drugs. The family should aid and understand the patient” (A* 59-*year*-*old female*)

“*My physician writes two prescriptions for me so I won’t be bothered every six months that I refer. By two prescriptions, I can buy drugs for three months. It’s great!” (A* 50-*year*-*old female*)

*“It is good if my father refers to physician once in a month. He feels he should listen to his physician who tells him if he does not observe, the illness may progress. The physician must inform his/her patient about the illness and its side-effects. In fact, my father irregularly has his drugs if not taking him to physician irregularly” (A 42-year-old member of one male patients’ family)*

*“The physician should support patients during the illness process. He/she should be good-tempered and communicates well with the patient. Strengthening the patients’ morale by the physician is very important. He/she should emphasize on the orderly use of the drugs. He/she should talk calmly. These mean the needed support!” (A* 63-*year*-*old female*).

*“The society and media should inform people about the prevention. During the education time and career, they should be instructed. Radio, TV and the newspapers should inform about diabetes and its side-effects. Also, insulin and glucose monitor test strips are expensive. The government should support by increasing the insurance coverage” (A* 58-*year*-*old male*)

##### Medication beliefs

Medication beliefs with three subthemes were identified as an influencing variable in the treatment adherence among this group. One of the subthemes was that the participants believed that anti-diabetic drugs does not affect the blood glucose control. Those participants with more negative beliefs about the treatment had more attempt in the medication use.

*“My father says he won’t get healed. I have diabetes. It makes no difference! To eat or not to eat the drugs does not affect diabetes!” (A 25-year-old member of one male patients’ family)*

*“I used pills for 8 years. Now, it has been two years that I use insulin. Using pills did not help me! I felt dizzy! When using insulin, it makes me feel better. But I do not know if it has any effect or not!” (A* 65-*year*-*old female*)

The second subtheme was that the participants believed that complementary therapies (e.g. healthy diet, physical activity and distillates and herbal drugs consumption) have more effectiveness than the anti-diabetic medications use.

*“Some prefer to eat diet foods so they do not need using pills. Having a food diet is more effective than pills” (A* 49-*year*-*old female*)

*“First I have to accept that I have diabetes. Then, I must have regimen and exercise. Last, drugs are effective” (A* 51-*year*-*old female*)

*“The traditional medicine doctor has given me some herbal capsules that I use. Distillates and herbal drugs are better than chemical drugs. I believe in them more than the pills” (A* 49-*year*-*old female*).

The third subtheme was that the participants preferred pills than the insulin injection. They have good feeling about the use of the pills. They do not have positive beliefs about the insulin and also it injection. From the viewpoints of some participants, eating pill is easier than the insulin injection. In addition, pill is considered as cheaper than the insulin. Some of the participants told that there was negative social stigma to insulin use in the family and community. They told that their family and community had negative insights about the insulin injection. They did not like to inject insulin in any situation, because they met with other people’s feelings of compassion. Some of the participants believed that the injection of insulin and its storage and conditions for keeping it outdoors was very difficult; they believed that if doctor prescribed insulin for them, it meant their disease had progressed and following the insulin injection diabetes complications (such as kidney loss) and ultimately death would appear.

*“I do not like to inject insulin. If using it, I feel my disease is progressing and I have not controlled it by the pills. Any way it is ampoule! Pills are not bothering than seeing the injection. Having an ampoule in my hands after each meal is agonizing for my children. It bothers me as well!”(A* 56-*year*-*old female*)

“*Carrying insulin on trips is difficult! It is hard to inject insulin in public! You should go to another room and inject there. Further, it is hard for me to learn how to inject!”(A* 60-*year*-*old male*)

*“Pills are cheaper, but insulin is expensive. I’m afraid of insulin, inflation and needle! Most of the patients say that injecting insulin shows the ultimate of the disease! These people have given a positive response to death! They wish for a relaxing death, so they try not to inject insulin” (A* 57-*year*-*old female*)

##### Cues to action

The majority of the study participants highlighted the importance of internal and external cues to medication adherence. The habit of taking medication and fear of diabetes-related complications were internal cues to regular prescribed drug using among the participants. In addition, setting the alarm to remind the time of medications use and the presence of medication in front of the eyes were reported as external cues to medication adherence by the participants.

*“Sometimes my father slams and does not eat his pills for some days. But he again fears and eats. The fear of foot scars, retinal hemorrhage or harm to kidneys can be as flips for the orderly use of the drugs. Those who have experienced the diabetes side-effects should be shown to others, so they use their drugs well” (A 25-year-old member of one male patients’ family)*

*“Since I have been using 9 pills per day for 12 years, I do not forget it like eating food. I am used to having it” (A* 57-*year*-*old male*)

“*I should keep my drugs in front of my eyes. Some set the alarm clock to remind them of eating the drugs” (A* 60-*year*-*old male*)

“*My wife calls me and reminds me of using the pills!* ” *(A* 53-*year*-*old female*).

## Discussion

The obtained results provided reputable information on the participants’ experiences of medication adherence. There appears to be several factors that affected the regular prescribed pharmacologic agents among a sample of Iranian patients with T2DM. Understanding these factors is required to develop tailored and effective efforts aimed at increasing the medication adherence among the patients with T2DM.

Perceived barriers with six subthemes including inadequate knowledge, situational influences, inadequate perceived threat, treatment characteristics, personality traits and medication cost are known as one of the participants’ experiences regarding the treatment adherence. The finding is consistent with those of Rubin who showed that the patient’s comprehension of the pharmacologic therapy regimen and its benefits, medication adverse effects, medication cost, regimen complexity and the patient’s emotional well-being may all influence the treatment adherence among the patients with T2DM [18]. Jeragh-Alhaddad et al. also showed that the lack of education/awareness about diabetes/medications and beliefs about medicines/diabetes can reduce the diabetic patients’ medication adherence [19]. Bailey and Kodack further reported that the poor adherence can be contributed to complex dosing regimens [20]. Considering the fact that almost majority of the barriers are resolvable by educating patients, courses of medication adherence should be developed and added in educational protocols for the diabetic patients. The content of the courses may consist of explanation about diabetes disease, diabetes’s acute and long-term complications, anti-diabetic drugs, its effectiveness and side-effects, the importance of regular medication use for the control of blood glucose level, simplifying the medication schedule, increasing active involvement and accountability of the patients in the process of medication use management effectively and helping the patient to overcome the medication adherence obstacles. For increasing the effectiveness of educational courses, using theory-based conseptual frameworks is suggested [21, 22].

In our study, some of the participants reported that medication beliefs may influence the treatment adherence. Some others believed that anti-diabetic drugs had no effect on diabetes outcomes. Almost a quarter of the participants (24%) perceived glucose-lowering medication as harmful chemicals which could have devastating effects on one’s health. Therefore, they preferred the use of complementary therapies such as herbs than medication. In addition, some of the participants stated that they had good feelings about the use of the pills such as Metformin, Glibenclamide and so on than injecting insulin. From their perspective, the pills were cheaper, easier to use and had less social stigma. Also, they mentioned that the injection of insulin and its storage was very difficult; their families had negative insights about the insulin injection; they believed that if doctor prescribed insulin for them, it meant their disease had progressed and following the insulin injection diabetes complications (such as kidney loss) would appear. This finding is consistent with the previous studies indicating that negative medication concerns could act as a barrier in the medication adherence [20, 23–26]. For example, Aflakseir showed that medication beliefs, such as concerns about the negative effects of anti-diabetic medicines, have an important role in poor adherence among diabetic patients [13]. Healthcare providers should identify patients’ perspective on medication beliefs and provide more effective information to modify misconceptions about the anti-diabetic medicines.

The authors also found evidence of poor compliance among the patients with low social support. In our study, family, doctor and community were three important sources of support for the patients regarding the medication adherence. The finding is consistent with those of other studies which highlighted the role of social support in adherence to diabetes treatment regimens [19, 27, 28]. As an example, Tiv et al. found that diabetic patients need family or social support, medical support, information on treatment and follow-up by a specialist for adherence to prescribed medications [27]. It’s worth mentioning that the study participants stated that good interaction of doctors with patients was a form of support. They expected that their doctors beside providing necessary information regarding diabetes, its consequences and the effectiveness of anti-diabetic medication get along well with the patients. In line with our findings, Abu Hassan et al. reported that doctors’ practical and emotional supports may help diabetic patients to accept insulin therapy [29]. This finding emphasized the importance of comprehensive support of the patients by healthcare providers, family and community as a whole in increasing the treatment adherence. This support was claimed achievable by educating family and policymakers to increase insurance coverage for the patients with chronic diseases and put pressure on mass media to design programs to raise the awareness of community about diabetes, and health care providers in terms of the effective communication with the patients.

Almost half the participants claimed that internal and external cues may impede or facilitate the blood glucose–lowering therapy adherence. Cues to action, which may be considered minor, are really important for the patient compliance and have been identified in other studies as well [30, 31]. It is suggested that innovative and cost-effective augment behavioral triggers such as medication reminders and challenge messages regarding the self-management be provided for the diabetic patients.

## Conclusions

Our study showed that multiple variables may affect the medication compliance of Iranian patients with T2DM. Some of the variables may be modified by patient education (such as increasing people awareness regarding diabetes) and others are influenced by social health policies (such as increasing insurance coverage and decreasing anti-diabetic drug cost). Identifying these variables may lead to the development of tailored interventions to increase the regular use of medicines in patients with T2DM. Health systems should consider medication adherence training to be added to the treatment protocols of diabetic patients.

### Strength and limitation

Although this study offered a number of key themes that could be useful in designing interventions aimed at further enhancing the medication adherence among the patients with T2DM in Iran, it suffers from some limitations. The first limitation was that our findings were derived from a qualitative research design. In fact, qualitative approach results cannot be generalized to larger populations. The second limitation was that data were collected from a sample of patients with T2DM who referred to the Institute of Endocrinology and Metabolism Research and Training Center of Firoozgar Academic Hospital and they were likely to be already connected to support services (e.g. education classes, peer support, medication). Performing similar studies in other racial/ethnic groups and geographic areas in Iran are suggested. Also, it is suggested that quantitative studies be performed to identify the factors influencing healthy lifestyle compliance among the diabetic patients in Iran. Developing and evaluating the adherence-enhancing interventions for the diabetic patients is also recommended.

## Abbreviation

T2DM: Patients with Type 2 Diabetes

## References

[1] Haghdoost AA, Rezazadeh-Kermani M, Sadghirad B, Baradaran HR (2009). Prevalence of type 2 diabetes in the Islamic Republic of Iran: systematic review and meta-analysis. East Mediterr Health J.

[2] American Diabetes Association (2011). Standards of medical care in diabetes—2011. Diabetes Care.

[3] Rubin RR (2005). Adherence to pharmacologic therapy in patients with type 2 diabetes mellitus. Am J Med Sci.

[4] Nathan DM, Buse JB, Davidson MB, Ferrannini E, Holman RR, Sherwin R (2006). Management of hyperglycemia in type 2 diabetes: a consensus algorithm for the initiation and adjustment of therapy: a consensus algorithm for the initiation and adjustment of therapy. Diabete Care.

[5] Delamater AM (2007). Improving patient adherence. Diabetes Care.

[6] Donnan PT, MacDonald TM, Morris AD (2002). Adherence to prescribed oral hypoglycemic medication in a population of patients with type 2 diabetes: a retrospective cohort study. Diabet Med.

[7] Farsaei S, Sabzghabaee AM, Zargarzadeh AH, Amini M (2011). Adherence to glyburide and metformin and associated factors in type 2 diabetes in Isfahan, Iran. Iran J Pharm Res.

[8] Farsaei S, Radfar M, Heydari Z, Abbasi F, Qorbani M (2014). Insulin adherence in patients with diabetes: risk factors for injection omission. Prim Care Diabetes.

[9] Jackson IL, Adibe MO, Okonta MJ, Ukwe CV (2015). Medication adherence in type 2 diabetes patients in Nigeria. Diabetes Technol Ther.

[10] Currie CJ, Peyrot M, Morgan CL, Poole CD, Jenkins-Jones S, Rubin RR (2012). The impact of treatment noncompliance on mortality in people with type 2 diabetes. Diabetes Care.

[11] Peyrot M, Barnett AH, Meneghini LF, Schumm-Draeger PM (2012). Insulin adherence behaviours and barriers in the multinational global attitudes of patients and physicians in insulin therapy. Diabet Med.

[12] Holt EW, Rung AL, Leon KA, Firestein C, Krousel-Wood MA (2014). Medication adherence in older adults: a qualitative study. Educ Gerontol.

[13] Aflakseir A (2012). Role of illness and medication perceptions on adherence to medication in a group of Iranian patients with type 2 diabetes. J Diabetes.

[14] Kassahun A, Gashe F, Mulisa E, Rike WA (2016). Nonadherence and factors affecting adherence of diabetic patients to anti-diabetic medication in Assela general hospital, Oromia region, Ethiopia. J Pharm Bioall Sci.

[15] Krass I, Schieback P, Dhippayom T (2015). Adherence to diabetes medication: a systematic review. Diabetes Care.

[16] Elo S, Kyngäs H (2008). The qualitative content analysis process. J Adv Nurs.

[17] Cypress BS (2017). Rigor or reliability and validity in qualitative research: perspectives, strategies, reconceptualization, and recommendations. Dimens Crit Care Nurs.

[18] Rubin RR (2005). Adherence to pharmacologic therapy in patients with type 2 diabetes mellitus. Am J Med Sci.

[19] Jeragh-Alhaddad FB, Waheedi M, Barber ND, Brock TP (2015). Barriers to medication taking among Kuwaiti patients with type 2 diabetes: a qualitative study. Patient Prefer Adherence.

[20] Bailey CJ, Kodack M (2011). Patient adherence to medication requirements for therapy of type 2 diabetes. Int J Clin Pract.

[21] Ebrahimipour H, Akerdi BJ, Solhi M, Esmaeli H (2015). Effect of educational intervention based on Self-Efficacy theory (SET) on behavior of prevention of HIV/ AIDS in high risk women. Iran J Obstet Gynecol Infertil.

[22] Solhi M, Saki M, Alimohammadi I, Haghani H (2012). Effect of health education based on BASNEF pattern on use of personal protective respiratory equipment in Ahvaz carbon block factory workers, 2009. IOH.

[23] Mann DM, Ponieman D, Leventhal H, Halm EA (2009). Predictors of adherence to diabetes medications: the role of disease and medication beliefs. J Behav Med.

[24] Sweileh WM, Zyoud SH, Abu Nab'a RJ, Deleq MI, Enaia MI, Nassar SM, et al. Influence of patients’ disease knowledge and beliefs about medicines on medication adherence: findings from a cross-sectional survey among patients with type 2 diabetes mellitus in Palestine. BMC Public Health. 2014;14(94). 10.1186/1471-2458-14-94. 10.1186/1471-2458-14-94 PMC3909379 24479638

[25] Clark M (2004). Adherence to treatment in patients with type 2 diabetes. J Diabetes Nurs.

[26] Vermeire E, Hearnshaw H, Rätsep A, Levasseur G, Petek D, van Dam H (2007). Obstacles to adherence in living with type-2 diabetes: an international qualitative study using meta-ethnography (EUROBSTACLE). Prim Care Diabetes.

[27] Tiv M, Viel J-F, Mauny F, Eschwège E, Weill A, Fournier C (2012). Medication adherence in type 2 diabetes: the ENTRED study 2007, a French population-based study. PLoS One.

[28] Gu L, Wu S, Zhao S, Zhou H, Zhang S, Gao M (2017). Association of social support and medication adherence in Chinese patients with type 2 diabetes mellitus. Int J Environ Res Public Health.

[29] Abu Hassan H, Tohid H, Mohd Amin R, Long Bidin MB, Muthupalaniappen L, Omar K (2013). Factors influencing insulin acceptance among type 2 diabetes mellitus patients in a primary care clinic: a qualitative exploration. BMC Fam Pract.

[30] Burner ER, Menchine MD, Kubicek K, Robles M, Arora S (2014). Perceptions of successful cues to action and opportunities to augment behavioral triggers in diabetes self-management: qualitative analysis of a mobile intervention for low-income Latinos with diabetes. J Med Internet Res.

[31] Finney Rutten LJ, Iannotti RJ (2003). Health beliefs, salience of breast cancer family history, and involvement with breast cancer issues: adherence to annual mammography screening recommendations. Cancer Detect Prev.

